# Congenital ventricular outflow tract obstructions in Boxer dogs: Results of a 17-year cardiovascular breed screening program in France (3126 dogs)

**DOI:** 10.1371/journal.pone.0285458

**Published:** 2023-05-11

**Authors:** Valérie Chetboul, Pauline Bernard, Peggy Passavin, Renaud Tissier

**Affiliations:** 1 Unité de Cardiologie d’Alfort, CHUV-Ac, École nationale vétérinaire d’Alfort, Maisons-Alfort, France; 2 Univ Paris Est Créteil, INSERM, IMRB, Créteil, France; 3 Unité de Médecine Interne, CHUV-Ac, École nationale vétérinaire d’Alfort, Maisons-Alfort, France; Vanderbilt University Medical Center, UNITED STATES

## Abstract

**Introduction:**

Ventricular outflow tract obstructions including aortic (AS) and pulmonic stenosis (PS) are the most common canine congenital heart diseases, with Boxer dogs being predominantly affected. This has led to the French Boxer club adopting a mandatory national control program against AS and PS. The objective of this retrospective study was to analyze the results of 17 years of this cardiovascular breed screening program (2005–2021).

**Materials and methods:**

The records of untreated and non-anesthetized adult Boxer dogs screened between 2005 and 2021 were retrospectively reviewed. All dogs underwent physical examination and standard transthoracic echocardiography with concomitant ECG tracing. All examinations were reviewed by one single board-certified specialist in cardiology.

**Results:**

Out of the 3126 dogs screened during the study period, 3001 dogs (female:male sex ratio = 2.2, median age [IQR] = 1.6 years [1.2–2.1]) were recruited for data analysis. A total of 218 operators were involved in the screening program. For most Boxer dogs (i.e., 93.8% for AS and 94.5% for PS), a single examination was required to obtain a definitive cardiac status, although most operators were non-specialist general practitioners. A left basilar systolic heart murmur was detected in all dogs with AS and PS, but also in 7.4% dogs free of heart diseases. A significantly higher proportion of the latter was detected when operators were board-certified specialists (P<0.001). Lastly, when comparing the start and the end of the breeding program, among dogs diagnosed with AS and PS (n = 364) in a French referral cardiology center, Boxer went from the 1^st^ affected breed by AS to the 3^rd^, and from the 3^rd^ affected breed by PS to the 6^th^.

**Conclusion:**

This 17-year screening program has experienced a strong involvement of veterinarians, breeders, and owners throughout France. This may have contributed to reduce AS and PS prevalence in Boxer dogs at the studied referral cardiology center.

## Introduction

Ventricular outflow tract obstructions (VOTO) including aortic (AS) and pulmonic stenosis (PS) are the most frequently canine congenital heart diseases (CHD) [1–6]. Congenital VOTO are defined by dynamic or fixed anatomic congenital obstructions to systolic flow from the left ventricle to the aorta for AS, and from the right ventricle to the pulmonary trunk for PS, each of them accounting for up to more than one third of all CHD in this species according to studies [1–4].

Canine subaortic stenosis (SAS) is the most common AS form in dogs, characterized by a fibrous or fibromuscular ring of tissue below the aortic valve resulting in left VOTO potentially leading to exercise intolerance, syncope, left-sided congestive heart failure, and even sudden death according to peak systolic Doppler-derived trans-stenotic pressure gradients (ΔP) [7, 8]. Valvular PS, more specifically type A valvular PS characterized by leaflet fusion and systolic doming, typically represents one of the most common forms of canine PS [1, 9–11]. Type B valvular PS (associating thickened leaflets with reduced valve leaflet motion and annulus hypoplasia) and intermediate valvular PS (combining type A and type B criteria) are less common [1, 9–11]. However, some breed specificities have been reported: for example, in the French Bulldog, PS is commonly severe and complex, with at least 2 obstructive lesions in most cases, and with a high incidence of pulmonary trunk hypoplasia and supravalvular stenosis [12]. Boxer dogs are known to be at high risk for developing several CHD including AS and PS [1, 2, 7, 8, 13–15]. Several studies demonstrated that canine breeds predisposed to SAS include Boxer, together with Newfoundland, German Shepherd, and Golden Retriever [7, 8]. Similarly, in several studies dedicated to canine PS, Boxer dogs represented the most commonly affected breed [11, 16]. Given the high prevalence and the suspected genetic origin of AS and PS in the Boxer breed, screening programs have been developed in Europe. The first one was set up in Italy in 1999 [13]. In 2005, the French Boxer club (entitled Boxer Club de France, and then Association Française du Boxer) set up a mandatory national cardiovascular screening program for detecting AS and PS, supervised by one single board-certified specialist, i.e., Diplomate of the European College of Veterinary Internal Medicine (Cardiology, VC).

The objective of the present retrospective study was to analyze the results of this 17-year French cardiovascular Boxer screening program (2005–2021).

## Materials and methods

### Animals

The records of 3126 breeder- and client-owned dogs that underwent the French mandatory cardiovascular screening program for detecting AS and PS in Boxer dogs between April 2005 and December 2021 were retrospectively reviewed. No ethical committee was required for this study owing to its retrospective epidemiological aspect and because it refers to a noninvasive clinical screening program for the French Boxer kennel club, and not at all to an experimental protocol used for teaching or research.

According to the official protocol, Boxer dogs had to be at least 12 months old, free of medication, and non-anesthetized for the procedure. Pedigrees and implanted microchips were checked and registered by operators involved in the program and both were re-checked by the French Boxer club. Dogs had to be examined by doctors in veterinary medicine (DVM), either board-certified specialists or general practitioners. As the latter were not cardiology specialists, in order to ensure data quality, all examinations were then reviewed and validated by one single board-certified specialist, i.e., Diplomate of the European College of Veterinary Internal Medicine (Cardiology, VC).

### Protocol

All Boxer dogs should undergo both complete physical examination and standard transthoracic echocardiography (M-mode, two-dimensional (2D) mode, and spectral Doppler) with concomitant electrocardiographic (ECG) tracing. Echocardiographic examinations could be performed with dogs in lateral recumbency or in a standing position. For all Boxer dogs, age, sex, body weight as well as operators’ name and their practice location were recorded. Heart murmurs were also recorded and categorized using a 6-level classification scheme [17]. Regarding Doppler examinations, peak aortic flow velocities were measured using continuous-wave Doppler mode from the left apical 5-chamber view or the subcostal view [9, 13]. Peak pulmonary flow velocities were also assessed using continuous-wave Doppler mode from the right or left short-axis view at the level of the aortic valve, as previously described [9, 13]. For each Doppler examination, all operators should provide good quality printed images showing at least two clearly defined Doppler curves with corresponding 2D images allowing to confirm that the Doppler cursor was optimally placed (i.e., along the predicted flow direction in the middle of each tested artery).

**Fig 1:**
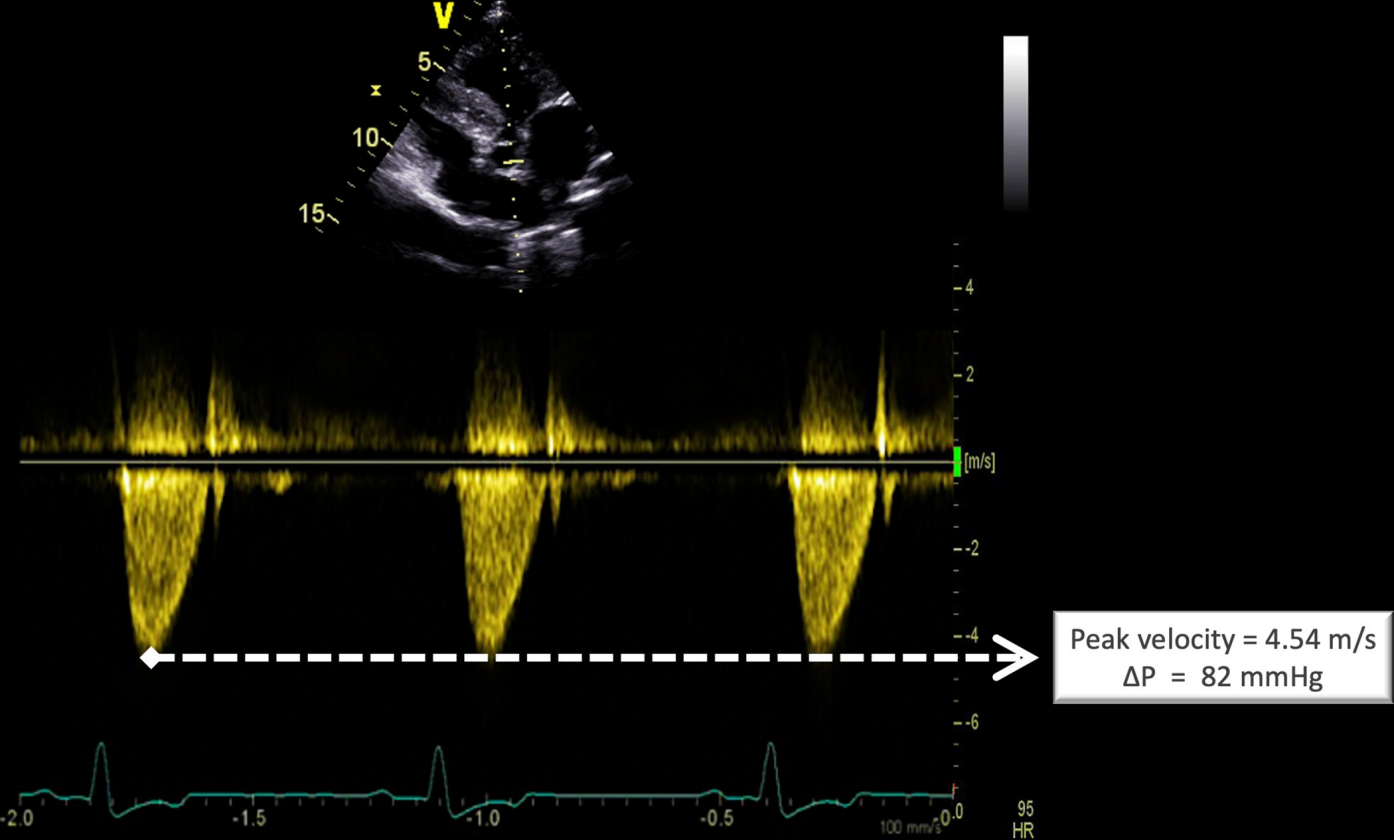
Representative continuous-wave Doppler echocardiographic view of a Boxer dog with severe congenital aortic stenosis showing an increased peak systolic aortic flow velocity recorded from the left apical 5-chamber view (peak systolic trans-stenotic pressure gradient (ΔP) = 82 mmHg).

The diagnosis of AS and PS was based on the following criteria (**Fig 1**): 1) systolic ejection heart murmur loudest in the left basilar area, and 2) peak aortic flow velocity > 2.6 m/s for AS and peak pulmonary flow velocity > 2.0 m/s for PS [18, 19]. The modified Bernoulli equation was then applied to calculate ΔP (in mmHg). Both AS and PS were considered as mild for ΔP ≤ 50 mmHg, moderate for ΔP > 50 and ≤ 80 mmHg, and severe for ΔP > 80 mmHg [18, 19].

The board-certified official reviewer rejected Doppler examinations if Doppler quality criteria were not fulfilled, e.g., poor to medium quality of 2D images, poorly defined Doppler curves, and incorrect placement of the Doppler cursor. In such cases, Doppler examinations were asked to be redone. In case of equivocal results, additional information was required by the official reviewer before a definitive diagnosis could be provided (e.g., pulsed-wave and color-flow Doppler examinations, and 2D images showing evidence of obstructive lesions).

Regarding the AS status, screened dogs were categorized after each examination by the official reviewer as “AS0” (absence of AS), “AS1”, “AS2”, or “AS3”, respectively corresponding to mild, moderate, and severe AS, as defined above. Dogs were also categorized regarding the PS status, as “PS0” (absence of PS), “PS1”, “PS2”, or “PS3”, respectively corresponding to mild, moderate, and severe PS, as also defined above. Lastly, the presence of other abnormalities (e.g., CHD, arrhythmias) was also recorded.

### Statistical analysis

For each screened Boxer dog, data of interest, i.e., main epidemiological features (age, body weight, sex), clinical (auscultation abnormalities), ECG findings, and Doppler variables (peak flow velocities and corresponding ΔP) were reported by the official reviewer in a spreadsheet (one per year). All spreadsheets (n = 17) were then gathered in one single file for the present study.

All statistics were performed by using a computer software (Addinsoft XLSTAT: statistical and data analysis solution, version 2020). Quantitative data were reported as median (interquartile range (IQR); minimal and maximal values) and percentage (%) when relevant. A Chi-squared test with Yates correction was used for qualitative variables. For all these analyses, the level of significance was set at P < 0.05.

## Results

### Epidemiological features

A total of 3126 Boxer dogs underwent the cardiovascular screening program between April 2005 and December 2021. After a final review, 54 dogs screened in 2005 and 71 in 2006 were excluded from further analysis owing to lack of clinical and echocardiographic data reported in the spreadsheets. Therefore, the study sample consisted of 3001 screened Boxer dogs (median age [IQR; minimum-maximum] = 1.6 years [1.2–2.1; 1.0–10.0] and body weight = 28.2 kg [26.0–32.0; 19.0–48.0]). Gender was known for 2945 out of the 3001 recruited Boxer dogs, i.e., 2036 females and 909 males corresponding to a female:male sex ratio of 2.2.

### Aortic and pulmonic stenosis status

Cardiac status of the study population regarding AS and PS is presented in **Table 1**. Out of the 3001 screened Boxer dogs, 2817/3001 (93.9%) and 2859/3001 (95.3%) were free of AS and PS, respectively. For the remaining dogs, 53/3001 (1.8%) and 30/3001 (1.0%) were affected by AS and PS, respectively. Out of these 83 dogs with congenital VOTO, 3 (3.6%) had combined AS and PS. Severity of AS and PS assessed by ΔP (mmHg) is shown in **Table 2**. For the remaining dogs, i.e., 131/3001 (4.4%) and 112/3001 (3.7%), Doppler examinations could not allow the official reviewer to definitively conclude on AS and PS status, respectively, because Doppler quality criteria were not fulfilled. For these dogs, the reviewer asked for new Doppler examinations. However, these requested re-examinations were not performed, avoiding a definitive cardiac status conclusion. Therefore, the definitive status regarding AS, PS, and combined AS and PS was obtained for 2870, 2889 and 2825 Boxer dogs, respectively.

**Table 1:** Distribution of the 3001 Boxer dogs included in the study out of the 3126 Boxer dogs that underwent the French cardiovascular breed screening program (2005–2021), according to their cardiac status regarding aortic stenosis (AS) and pulmonic stenosis (PS) for which information was available.

Cardiac status	Sub-classes					
**Aortic stenosis**	**AS0**	**AS1**	**AS2**	**AS3**	**ASNC**	**Total**
*Number of Boxer dogs*	2817	45	5	3	131	3001
*Percentage (%)*	93.9	1.5	0.2	0.1	4.4	100
**Pulmonic stenosis**	**PS0**	**PS1**	**PS2**	**PS3**	**PSNC**	**Total**
*Number of Boxer dogs*	2859	25	4	1	112	3001
*Percentage (%)*	95.3	0.8	0.1	0.03	3.7	100

AS0, AS1, AS2, AS3: dogs with no AS, mild, moderate, and severe AS, respectively.

PS0, PS1, PS2, PS3: dogs with no PS, mild, moderate, and severe PS, respectively.

ASNC and PSNC: dogs with nonconclusive Doppler examinations of aortic and pulmonary flows, respectively.

**Table 2:** Peak systolic velocity (Vmax, m/s) and Doppler-derived pressure gradient (ΔP, mmHg) in Boxer dogs for which the board-certified specialist (VC) in charge of the French cardiovascular breed screening program (2005–2021) could conclude to a definitive cardiac status regarding aortic stenosis (n = 2527) and pulmonic stenosis (n = 2542) out of the 3126 Boxer dogs that underwent the French cardiovascular breed screening program (2005–2021) and for which corresponding information was available. Dogs with other heart diseases were excluded from the analysis.

Boxer’s category	Number of Boxer dogs	Median ΔP (mmHg) (interquartile range; minimum-maximum)	Median Vmax (m/s) (interquartile range; minimum-maximum)
**AS0**	2494	13 (11–16; 3–26)	1.82 (1.65–1.98; 0.91–2.55)
**AS1**	30	34 (30–37; 23–46)	2.91 (2.76–3.0; 2.41–3.39)
**AS2**	2	62 (60–63; 59–65)	3.93 (3.88–3.98; 3.83–4.03)
**AS3**	1	82	4.54
**PS0**	2522	8 (6–10; 2–17)	1.38 (1.25–1.54; 0.61–2.0)
**PS1**	17	38 (27–45; 18–50)	3.06 (2.6–3.34; 2.14–3.54)
**PS2**	2	57 (57–57; 56–57)	3.77 (3.75–3.77; 3.75–3.78)
**PS3**	1	85	4.6

AS0, AS1, AS2, AS3: dogs with no AS, mild, moderate, and severe AS, respectively.

PS0, PS1, PS2, PS3: dogs with no PS, mild, moderate, and severe PS, respectively.

Out of the 50 Boxer dogs diagnosed with AS and free of PS, 32/50 (64.0%) were females and 18/50 (36.0%) were males. Out of the 27 Boxer dogs diagnosed with PS and free of AS, 16/27 (59.3%) were females and 11/27 (40.7%) were males. No significant sex predisposition was found for both AS and PS (P = 0.48 and P = 0.34, respectively).

Distribution of stenosis lesions (i.e., valvular, subvalvular, or supravalvular) was available for 34/53 dogs diagnosed with AS and 14/30 dogs with PS. A single obstructive lesion was present in almost all Boxer dogs with congenital VOTO, i.e., 34/34 (100%) for AS and 13/14 (92.9%) for PS, with only one affected by combined valvular and supravalvular PS. Subvalvular AS was the most common AS stenotic lesion (24/34, 70.6%), followed by valvular (9/34, 26.5%), and supravalvular (1/34, 2.9%). Out of the 13 PS dogs with single stenotic lesion, valvular PS (9/13, 69.2%) was the most common PS type followed by supravalvular PS (3/13, 23.1%) and subvalvular PS (1/13, 7.7%).

### Cardiac abnormalities other than congenital VOTO

Out of the 3001 screened Boxer dogs, 307 (10.2%) were found affected by at least one CHD other than AS and PS, i.e., mild mitral valve dysplasia (166/3001; 5.5%), atrial septal defect (120/3001; 4.0%), mild aortic valve regurgitation (91/3001; 3.0%) owing to bicuspid and quadricuspid aortic valve in one (1/3001; 0.03%) and seven (7/3001; 0.2%) dogs respectively, and tricuspid valve dysplasia (10/3001; 0.3%). Electrocardiographic abnormalities were reported in 15/3001 Boxer dogs (0.5%): second-degree atrioventricular block (n = 2), and ventricular premature complexes (n = 13). None of these 15 Boxer dogs with arrhythmias were affected by AS or PS. Conversely, 7/13 dogs with ventricular premature beats had either mitral valve dysplasia, (n = 5) or atrial septal defect (n = 2), and one Boxer with second-degree atrioventricular block had concomitant mitral valve dysplasia and atrial septal defect.

### Operators involved in the screening program

**Fig 2:**
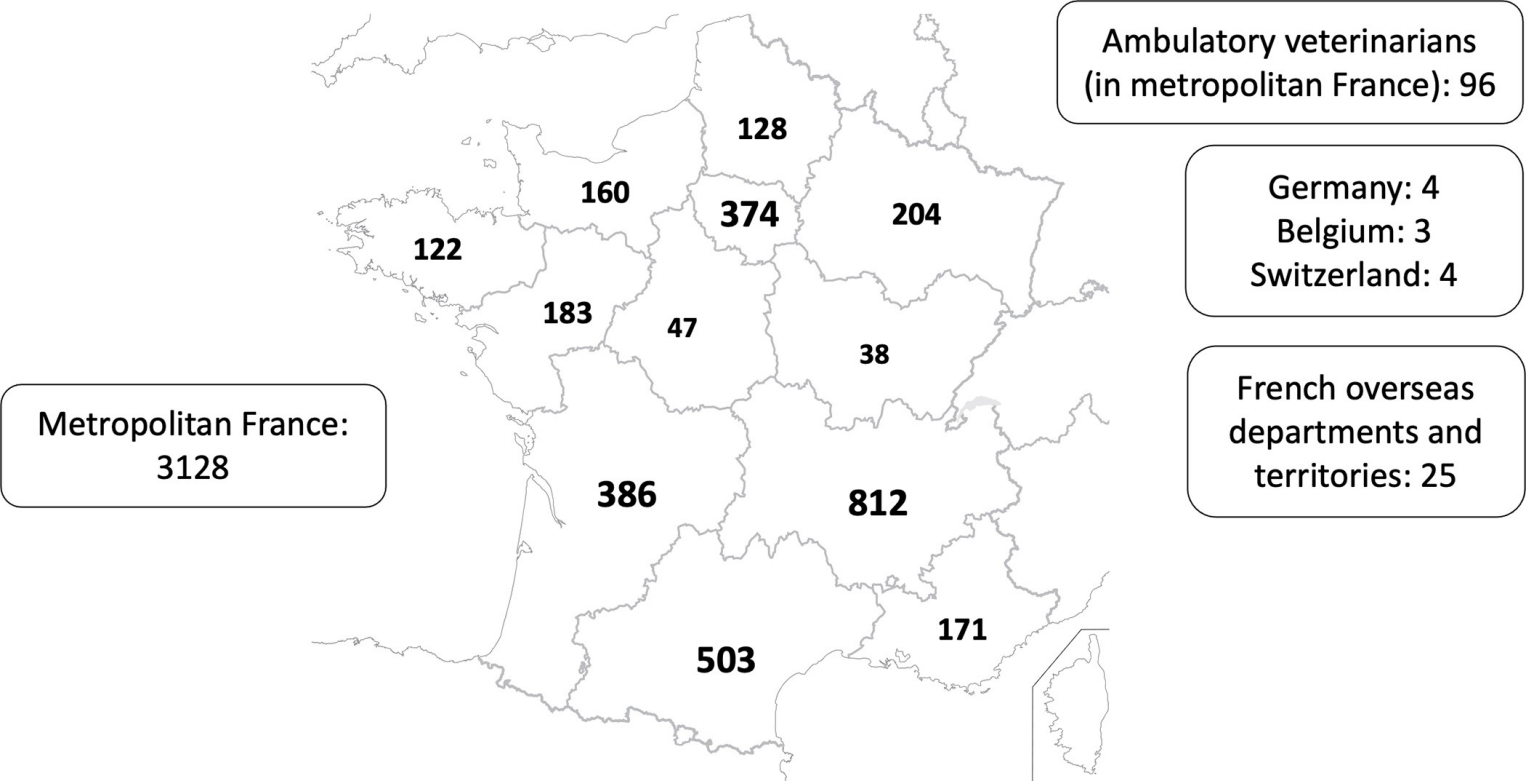
Geographical distribution of echocardiographic examinations (n = 3260) performed in 218 screening veterinary centres on 2995 Boxer dogs for which information was available (i.e., a total of 3269 echocardiographic examinations were performed on 3001 Boxer dogs, and for 9 of which information regarding the veterinary centre location was lacking). Map of France from the Institut National de l’Information Géographique et Forestière, France (https://ign.fr/institut/ressources-pedagogiques).

A total of 218 operators were involved in the cardiovascular screening program, representing 3269 examinations on 3001 Boxer dogs, 2927 of which (97.5%) were examined in a standing position, 73 (2.4%) in lateral recumbency, and 1 (0.03%) in both positions. Nine of these 218 operators (4.1%) were board-certified specialists, while 209 (95.9%) were general practitioners (corresponding to 568 and 2701 examinations, respectively). Out of the 3260/3269 screening examinations for which geographical information was available (**Fig 2**), nearly all (i.e., 3224/3260, 98.9%) were performed in metropolitan France either in veterinary centers (n = 3128/3224, 97.0%) or by ambulatory veterinarians (n = 96/3224, 3.0%).

Among the 2870 and 2889 Boxer dogs with a definitive cardiac status for AS and PS respectively, numbers of screening examinations needed to definitely conclude the cardiac status are presented in **Table 3**. For most Boxer dogs (i.e., 93.8% for AS and 94.5% for PS), a single examination allowed to obtain a definitive cardiac status. The median (IQR; minimum-maximum) number of examinations required to conclude to the definitive cardiac status was 1 (1–1; 1–3) for AS and 1 (1–1; 1–6) for PS.

**Table 3:** Number of Doppler examinations (i.e., 1, 2, or ≥3) that allowed the board-certified specialist (VC) in charge of the French cardiovascular breed screening program (2005–2021) to conclude to a definitive cardiac status regarding aortic stenosis (AS) and pulmonic stenosis (PS) (n = 2870 for AS and n = 2889 for PS out of the 3126 Boxer dogs that underwent the screening program and for which corresponding information was available).

**Stenosis status**	*Aortic stenosis category (number of Boxer dogs)*				**Total of Boxer dogs**	**Percentage (%)**
	**AS0**	**AS1**	**AS2**	**AS3**		
**Number of examinations required to conclude**						
1	2650	37	2	3	2692	93.8
2	151	6	2	0	161	5.6
≥3	14	2	1	0	17	0.6
**Total**	2817	45	5	3	2870	100
**Stenosis status**	*Pulmonic stenosis category*				**Total of Boxer dogs**	**Percentage (%)**
	**PS0**	**PS1**	**PS2**	**PS3**		
**Number of examinations required to conclude**						
1	2702	23	4	1	2730	94.5
2	141	2	0	0	143	4.9
≥3	16	0	0	0	16	0.6
**Total**	2859	25	4	1	2889	100

AS0, AS1, AS2, AS3: dogs with no AS, mild, moderate, and severe AS, respectively.

PS0, PS1, PS2, PS3: dogs with no PS, mild, moderate, and severe PS, respectively.

No statistical difference was found (P = 0.11) regarding the number of screening examinations required to obtain a definitive cardiac status for AS using a single examination between board-certified specialists (n = 9) and general practitioners (n = 209), i.e., respectively 498/522 (95.4%) and 2194/2348 (93.4%). Conversely, the number of screening examinations required to obtain a definitive cardiac status for PS using a single examination was significantly lower (P = 0.027) for board-certified specialists than for general practitioners, i.e., respectively 508/526 (96.6%) and 2222/2363 (94.0%).

### Cardiac auscultation

Out of the 2485 Boxer dogs with a definitive AS and PS cardiac status, not suffering from other CHD than AS and PS and for which auscultatory information was available, auscultation was normal for 2223/2485 dogs (89.5%) while a left basilar systolic heart murmur (LBSM) was detected in 230/2485 Boxer dogs (9.3%) either healthy or affected by AS and/or PS (**Table 4**), with a median (IQR; minimum-maximum) LBSM grade of 1/6 (1/6-2/6; 1/6-4/6). A LBSM was detected in all Boxer dogs with AS and PS, and also in 181/2436 (7.4%) Boxer dogs free from AS, PS, and other CHD (**Table 4**). For the latter, the LBSM grade was low (i.e., 1/6 or 2/6) for most dogs (i.e., 176/181, 97.2%). The proportion of low grade LBSM (i.e., grades 1/6 and 2/6) detected in apparently healthy Boxer dogs was significantly higher for board-certified specialists than for general practitioners (P < 0.001).

**Table 4:** Characteristics of the left basilar systolic heart murmur (LBSM) detected in 230/2485 Boxer dogs either healthy (AS0 and PS0) or affected by aortic and/or pulmonic stenosis out of the 3126 Boxer dogs that underwent the French cardiovascular breed screening program (2005–2021) and for which corresponding information was available. Dogs with other heart diseases than aortic stenosis and pulmonic stenosis were excluded from the analysis.

Boxer dog’s category		Number of Boxer dogs	Number (%) of Boxer dogs with a LBSM	Number of Boxer dogs with LBSM grade available	Median grade(interquartile range; minimum-maximum)	Number of Boxer dogs			
						Grade 1/6	Grade 2/6	Grade 3/6	Grade 4/6
**No aortic and pulmonic stenosis**	*AS0 and PS0*	2436	181 (7.4%)	181	1 (1–2; 1–3)	111	65	5	0
**Single stenosis**	*AS1 and PS0*	26	26 (100%)	24	2 (2–3; 1–4)	5	12	5	2
	*AS2 and PS0*	2	2 (100%)	2	2.5 (2.3–2.8; 2–3)	0	1	1	0
	*AS3 and PS0*	1	1 (100%)	1	-	0	0	1	0
	*PS1 and AS0*	17	17 (100%)	16	3 (2–3; 1–4)	2	4	7	3
	*PS2 and AS0*	1	1 (100%)	1	-	0	1	0	0
	*PS3 and PS0*	1	1 (100%)	1	-	0	0	1	0
**Double arterial stenosis**	*AS1 and PS2*	1	1 (100%)	1	-	0	1	0	0

AS0, AS1, AS2, AS3: dogs with no AS, mild, moderate, and severe AS, respectively.

PS0, PS1, PS2, PS3: dogs with no PS, mild, moderate, and severe PS, respectively.

### Proportion of dogs with AS and PS: Comparison between the start and the end of the breeding program

A French referral cardiology center (Alfort Cardiology Unit, France) was chosen for evaluating canine breeds affected by AS and PS over time. During the study period (2005 to 2021), a total of 364 dogs of 67 different breeds were diagnosed with either AS (n = 110, 31 breeds) or PS (n = 254, 53 breeds, including 2 dogs with both AS and PS) in this center. Regarding AS, the five most commonly affected breeds were Boxer (32/110, 29.1%), followed by Bull Terrier (12/110, 10.9%), Golden Retriever (10/110, 9.1%), Newfoundland (6/110, 5.4%), and German Shepherd (6/110, 5.4%). Between 2005 and 2009 (i.e., start of the cardiovascular breed screening program), out of the 42 dogs diagnosed with AS from 17 different breeds, the Boxer was by far the most commonly affected breed (18/42, 42.9%), followed by the Bull Terrier (3/42, 7.1%) and Golden Retriever (3/42, 7.1%). Conversely, between 2017 and 2021 (i.e., end of the cardiovascular breed screening program), out of the 50 dogs diagnosed with AS from 20 different breeds, the Boxer was ranked 3^**rd**^ (5/50, 10%) *ex æquo* with the White Swiss Shepherd and American Bully, behind the Golden Retriever (7/50, 14%) and Bull Terrier (6/50, 12%) respectively ranked 1^**st**^ and 2^**nd**^.

Similarly, between 2005 and 2021, regarding PS, the five most commonly affected breeds were the French Bulldog (77/254, 30.3%), followed by the English Bulldog (14/254, 5.5%), American Staffordshire Terrier (13/254, 5.1%), Boxer (12/254, 4.7%), and Golden Retriever (11/254, 4.3%). Between 2005 and 2009 (i.e., start of the cardiovascular breed screening program), out of the 85 dogs diagnosed with PS from 26 different breeds, the Boxer was ranked 3^**rd**^ *ex æquo* with the English Bulldog (6/85, 7.1%), behind the French Bulldog (27/85, 31.8%) and Bull terrier (7/85, 8.2%), respectively ranked 1^**st**^ and 2^**nd**^. Conversely, between 2017 and 2021 (i.e., end of the cardiovascular breed screening program), out of the 122 dogs diagnosed with PS from 37 different breeds, the Boxer was ranked 6^**th**^ (3/122, 2.5%) *ex æquo* with several breeds (American Bully, German Shepherd, English Bulldog, Cane Corso, Pinscher, and Yorkshire), behind the French Bulldog (37/122, 30.3%), American Staffordshire Terrier (8/122, 6.6%), Golden Retriever (7/122, 5.7%), Spitz (6/122, 4.9%), and Swiss Shepherd (5/122, 4.1%).

## Discussion

Owing to the high predisposition of Boxer dogs to CHD, and more specifically congenital VOTO, reported in the 2000’s [10, 11, 14], a cardiovascular screening program using both physical examination and echocardiography was set up in France in 2005, with the objective to decrease AS and PS prevalence over time in the Boxer breed. The present study provides relevant information on this 17-year program with more than 3000 tested dogs representing, to the best of our knowledge, the largest reported Boxer dog population in veterinary cardiology.

This French cardiovascular screening program is mandatory since 2005 for any Boxer dog intended for breeding. Therefore, our study sample is representative of the Boxer breeding population in France rather than the whole French Boxer population. This probably explains that the study sample included more than twice females than males with a majority of young adult animals (75% of dogs were under than 2.1 years old with a median age of 1.6 years only). As previously reported [13], the body weight range was wide (19.0 to 48.0 kg, with a median value of 28.2 kg).

One of the main features of this 17-year program is the large number of screened Boxer dogs, i.e., 3126 dogs, 3001 of which were recruited for data analysis. This could be achieved thanks to a strong involvement of French veterinarians, with a total of 218 operators included in the program. Interestingly, very few of them (4.1%) were board-certified specialists, as the latter are uncommon in France (only 9 during the study period), the large majority (95.9%) being non-specialist general practitioners. Limiting the protocol to board-certified specialists would have considerably reduced the number of screened dogs, and thus the impact of this screening protocol, as nearly 82.6% of examinations (2701/3269) were performed by non-specialist general practitioners. This explains the decision to involve the latter in the screening program, with supervision of all examinations by a single official board-certified specialist who could conclude to a definitive cardiac status for most Boxer dogs (i.e., 93.8% for AS and 94.5% for PS) using one single examination only, less than 1% of dogs requiring ≥ 3 examinations. The advantage and feasibility of involving general practitioners with appropriate echocardiographic training and experience has recently been emphasized in small animal cardiology for standard examinations [20–22]. For example, focused cardiac ultrasound performed by non-specialist general practitioners has been demonstrated to increase the detection of occult heart diseases in cats, although the diagnostic ability increases with disease severity [21]. Nevertheless, in the present study, board-certified specialists detected significantly more innocent heart murmurs (i.e., low grade LBSM in Boxer dogs free of heart diseases [23]) than general practitioners. These results are consistent with previous studies showing that auscultatory findings depend on observer’s experience [24–26]. In one study, inter-observer variation in the detection and grading of low-grade heart murmurs was investigated in Boxer dogs, and inter-observer agreement was demonstrated to be positively correlated to the level of experience, with most experienced observers being able to better detect soft murmurs than others [25]. Soft heart murmurs are frequently reported in healthy Boxer dogs, in up to more than 50% in some studies [10, 27]. In the present report, a soft LBSM (grade 1 or 2 for 97.6% of cases, with a maximum grade of 3) was also found in apparently healthy Boxer dogs, however with a lower prevalence than in other reports (7.4%). This result may be in part explained by the high proportion of general practitioners involved in the program, as compared to board-certified specialists [24–26]. Innocent ejection murmurs in Boxer dogs may be related to increased aortic flow velocity (> 2 m/s) owing to exaggerated sympathetic tone and anatomic variations of the left ventricular outflow tract and the aorta [15, 18, 27]. Phonocardiographic characteristics (e.g., duration of murmur frequency > 200 Hz) can be helpful in Boxer dogs for differentiation between these nonpathological murmurs and murmurs caused by mild AS, the latter being frequently reported in this canine breed [13, 28]. However, these two types of low-intensity murmurs cannot be distinguished using a standard stethoscope [28, 29]. This limitation of cardiac auscultation illustrates the need of echocardiographic examination to differentiate nonpathological heart murmurs from murmurs caused by mild AS and confirms the practical interest of the present cardiovascular screening program to detect congenital VOTO in Boxer dogs.

Although the present study was conducted on a large number of Boxer dogs, AS and PS prevalence in France in this canine breed cannot be assessed from the presented data, because the study sample is not representative of the whole French Boxer population as explained above. Nevertheless, findings from the 83 Boxer dogs with congenital VOTO were consistent with previous reports, with a large majority of single obstructive lesions (97.9%), and predominance of subvalvular AS and valvular PS, representing 70.6% and 69.2% of all AS and PS, respectively [1, 10, 13, 14]. Combined AS and PS was diagnosed in 3.6% of the 83 dogs with congenital VOTO. Such combined obstructive lesions have already been reported in the Boxer breed in up to 25% of cases [10, 13]. Lastly, a predisposition of male Boxer dogs for both AS and PS has been suggested by some authors [13] but was not found in the present study. However, our study sample including a majority of females intended for breeding could have biased this result.

Although left and right VOTO represent the most commonly diagnosed CHD in the Boxer breed, other congenital abnormalities are also reported [6, 13, 14]. Similarly, in the present study, 10.2% of the 3001 screened Boxer dogs were affected by at least one CHD other than AS and PS, the two most common being mild mitral valve dysplasia (54.1%) and atrial septal defect (39.1%). These results are consistent with those from other reports showing that mitral valve dysplasia and atrial septal defect (ostium secundum type) are common in the Boxer breed [6, 14]. In one study including 113 atrial septal defects in dogs [6], 40 canine breeds were represented, with Boxer (31.9%) predominating, followed by Cavalier King Charles (7.1%), German Shepherd (5.3%) and Labrador Retriever (5.3%). Comparison with a reference population of 30174 dogs confirmed a significant predisposition of Boxer dogs to atrial septal defect (odds ratio [Confidence interval] of 15.28 [10.24–22.84], P <0.05) [6].

In our study, all screened dogs underwent ECG tracings concomitantly with standard transthoracic echocardiography. Interestingly, electrocardiographic abnormalities including mostly ventricular premature beats were reported in only 0.5% and 0.4% of the 3001 tested Boxer dogs, none of which being affected by congenital VOTO. Similarly, records of 1283 screened Boxer dogs in Italy showed a low prevalence of arrhythmias, with ventricular premature beats in 0.08% dogs only [13]. However, 24-hour Holter studies are needed to better assess the prevalence of arrhythmias in the French Boxer breed population.

Among dogs diagnosed with AS and PS (n = 364) in a French referral cardiology center, when comparing the start (between 2005 and 2009) and the end (between 2017 and 2021) of the breeding program, Boxer went from the 1^**st**^ affected canine breed by AS (42.9%) to the 3^**rd**^ (10%), and from the 3^**rd**^ affected breed by PS (7.1%) to the 6^**th**^ (2.5%). This decreased proportion of Boxer dogs with congenital VOTO (minus 67% for combined AS and PS) may be at least in part related to the beneficial impact of the cardiovascular breeding program, all the more because according to French Boxer club official data, births of Boxer dogs in France decreased in much lesser extent, i.e., minus 17% between the start and the end of the program (mean of 2325 and 1936 births/year, respectively [30]). Similarly, a mandatory cardiovascular screening program in Boxer dogs in Italy, using both physical examination and echocardiography, showed a lower overall prevalence of congenital VOTO over a 6-year period [13].

Since 2008, the recommendation of the French Boxer club is to avoid breeding of Boxer dogs with both moderate and severe AS. Breeding of dogs with mild AS is not advised or should be done only with AS0 dogs in exceptional cases. No specific recommendation has been made regarding PS. Considering the results of the present study, further recommendation could be formulated, e.g., AS1 Boxer dogs should not be used for breeding, with similar recommendations for PS.

This study presents several limitations. Firstly, the official board-certified specialist in charge of the program could not validate data from physical examination and owing to the large number of general practitioners involved in the screening, the proportion of dogs with soft heart murmurs may have been underestimated. For the same reasons, the proportion of other CHD than AS and PS (e.g., mitral valve dysplasia and atrial septal defect) may also have been underestimated. Additionally, left and right ventricular free wall thicknesses were used by the official board-certified specialist in charge of the program to validate echocardiographic and Doppler examinations. However, these imaging variables were not included in the data base and therefore were not available for the present retrospective study. Lastly, the board-certified official reviewer could not conclude to a definitive cardiac status owing to poor to medium quality of Doppler examinations. However, this represented 4.4% and 3.7% only of all screened dogs for AS and PS, respectively.

In conclusion, this 17-year cardiovascular screening program set up in the Boxer breed has experienced a strong involvement of veterinarians, breeders, and owners throughout France, with a large number of tested dogs (more than 3000). The majority of them (> 90%) were free of AS and PS, and were therefore officially allowed for breeding, which may have contributed to improve cardiac health of this breed, as shown in the referral center.
